# Supplementary material for: Real-Time Prediction of Lower-Limb Joint Kinematics, Kinetics, and Ground Reaction Force Using Wearable Sensors and Machine Learning
Source: Sensors (Basel). 2026 Sep 17;26(18):5885. doi: 10.3390/s26185885 (PMC13611085; doi:10.3390/s26185885)
Supplement: Supplementary file 1 [file sensors-26-05885-s001.zip › sensors-4492031-supplementary.pdf]

Real-Time Prediction of Lower Limb Joint Kinematics, Kinetics, and Ground Reaction Force using Wearable Sensors and Machine Learning

Josée Mallah [1], Yu Zhu [1,2], Kailang Xu [1], Gurvinder S. Virk [3,4], Shaoping Bai [5], and Luigi G. Occhipinti [1,6] *

[1] Electrical Engineering Division, Department of Engineering, University of Cambridge, Cambridge CB3 0FA, United Kingdom
[2] Department of Electronic Systems, Aalborg University, Aalborg 9220, Denmark
[3] Endoenergy Systems Ltd., Cambridge, United Kingdom
[4] Huzhou Wuxing District Intelligent Robot Innovation Research Institute, China
[5] Department of Materials and Production, Aalborg University, Aalborg 9220, Denmark
[6] Wolfson School of Mechanical, Electrical and Manufacturing Engineering, Loughborough University, LE11 3TU, Loughborough, United Kingdom
* Correspondence: l.g.occhipinti@lboro.ac.uk

Supplementary Materials

Supplementary Figures S1-S5

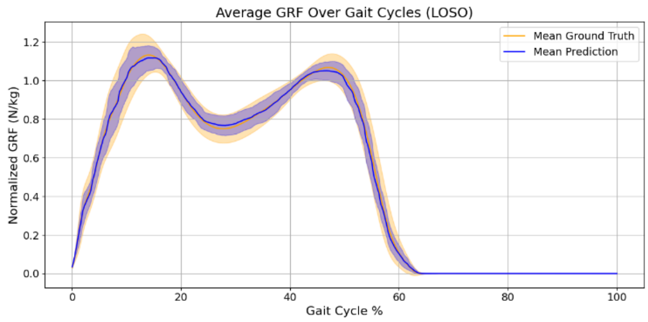

Figure S1: Average GRF over the gait cycle duration - predictions vs ground truth in inter-subject mode (section 3.1).

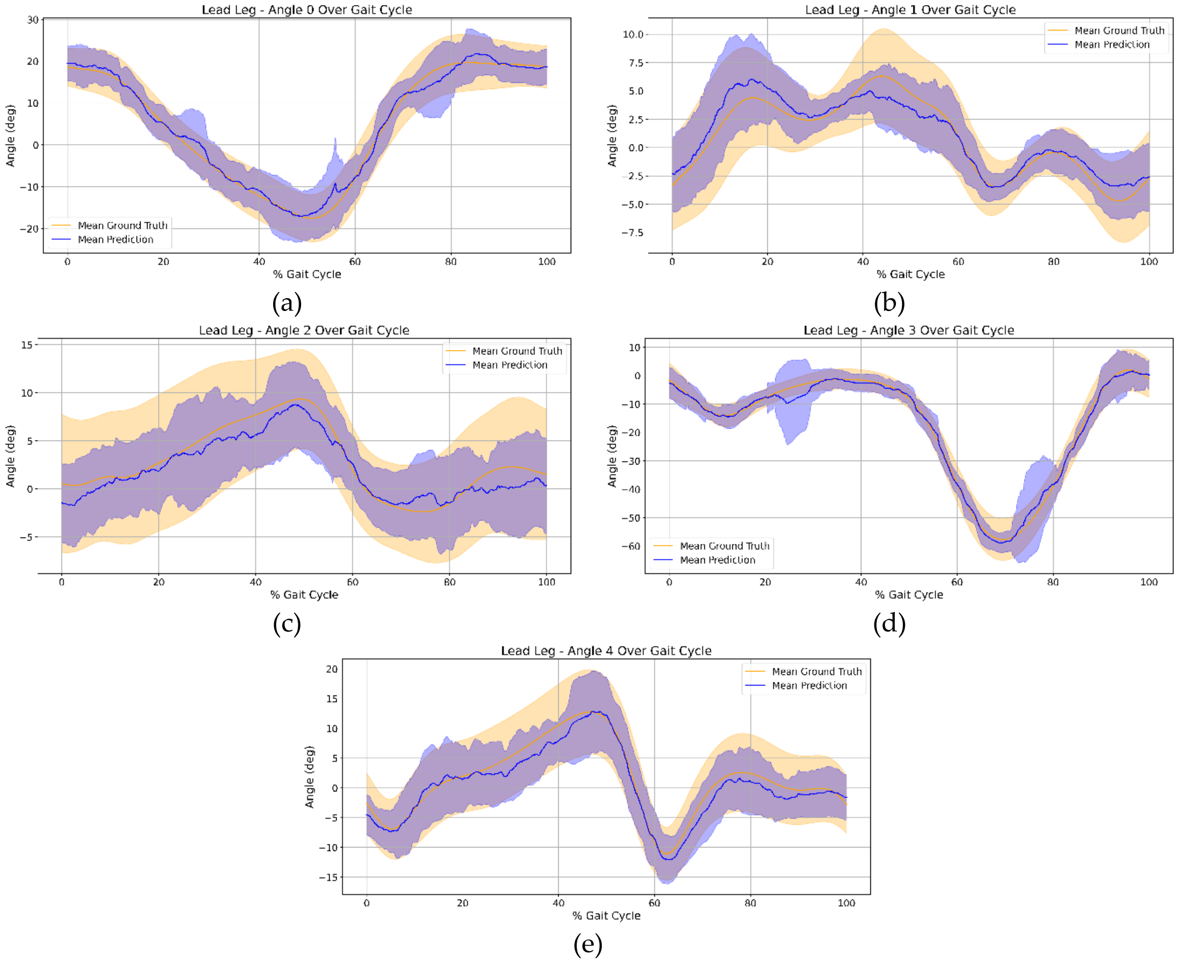

Figure S2: Angle model W6 – Ensemble averages and standard deviation of the ground truth versus predicted joint moments for the hip flexion (a), adduction (b), and rotation (c), knee flexion (d), and ankle flexion (e) at the leading feet, in inter-subject mode (section 3.2).

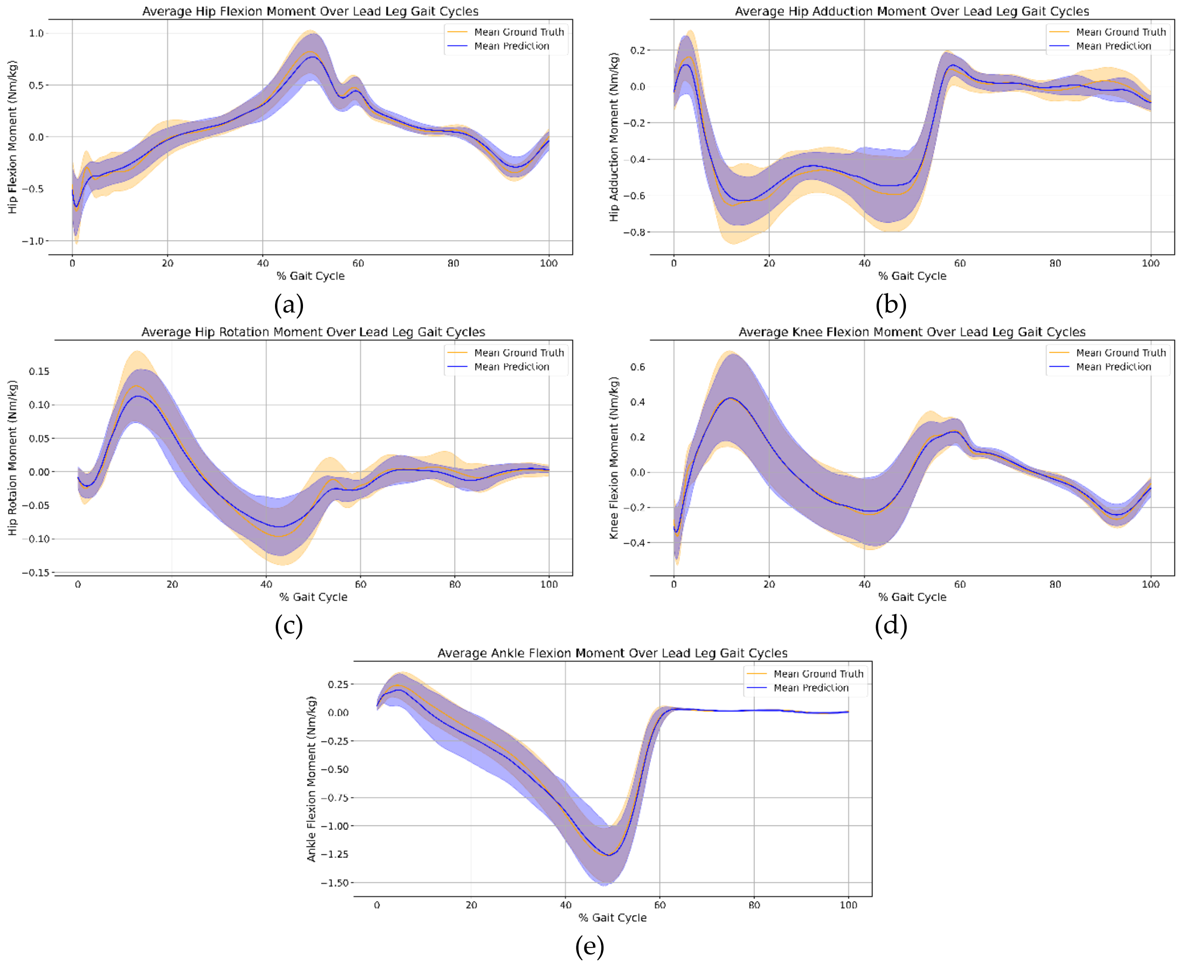

Figure S3: Ensemble averages and standard deviation of the ground truth versus predicted joint moments for the hip flexion (a), adduction (b), and rotation (c), knee flexion (d), and ankle flexion (e) at the leading leg in inter-subject mode (section 3.3).

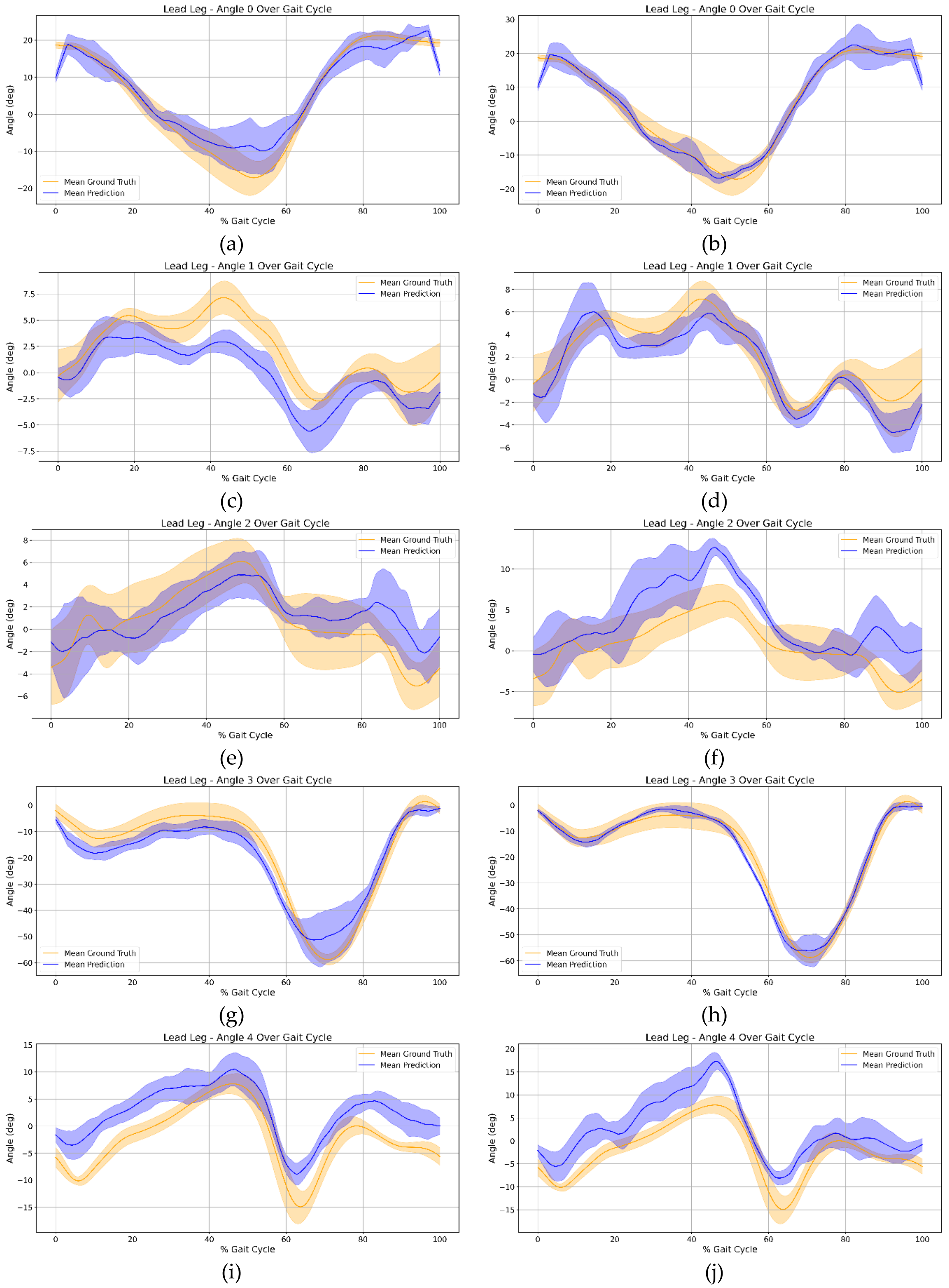

Figure S4: Predictions (filtered) vs ground truth data for the hip flexion (a, b), adduction (c, d), and rotation (e, f), and knee (g, h) and ankle (i, j) flexion angles using angle models W4 (a, c, e, g, i) and W5 (b, d, f, h, j) and model chaining (offline, section 3.4).

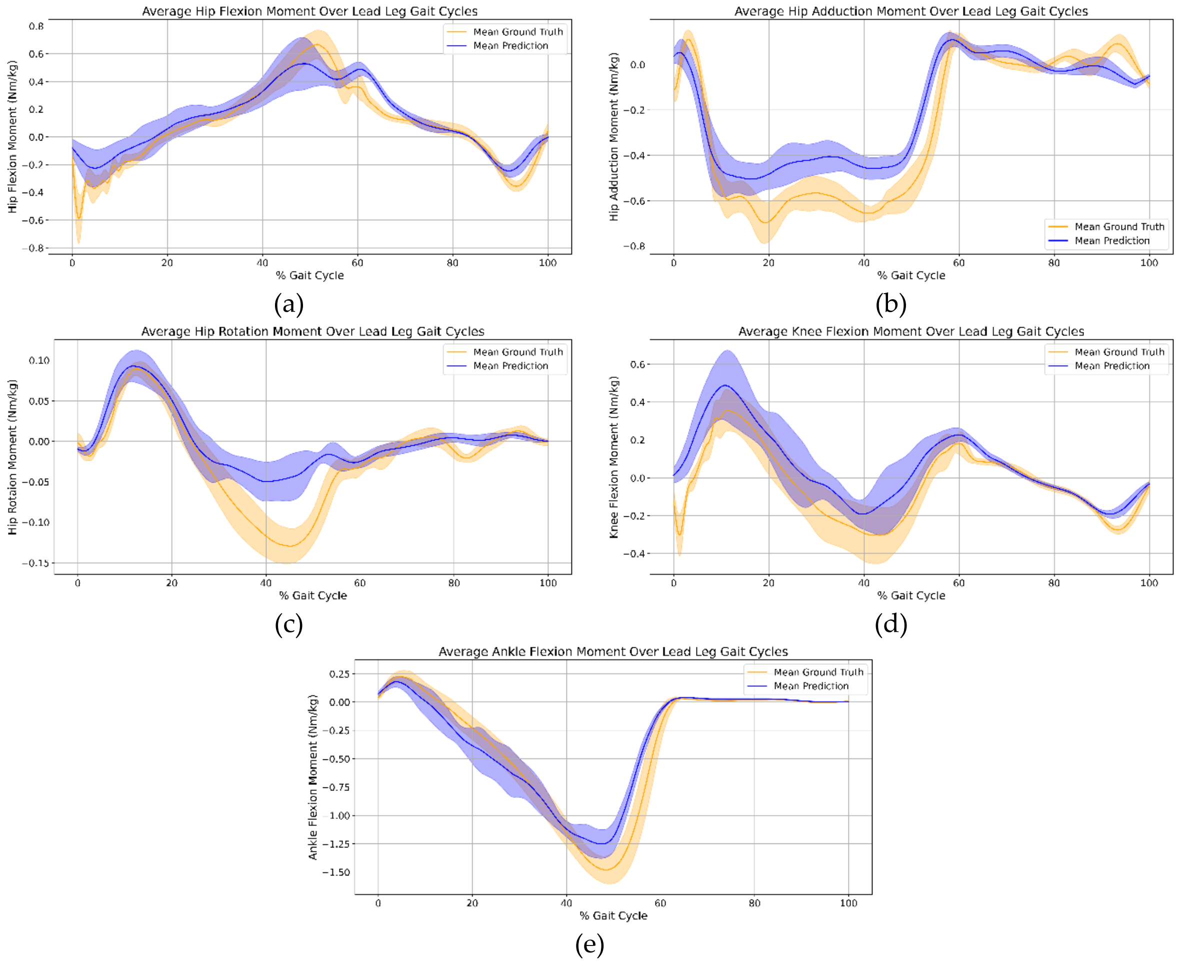

Figure S5: Predictions (filtered) vs ground truth data for the hip flexion (a), adduction (b), and rotation (c), and knee (d) and ankle (e) flexion moments using angle data from model W4 and model chaining (offline, section 3.4).
